# The diagnostic significance of pupillary reflex pathways: insights from classical examination and advanced pupillometry

**DOI:** 10.3389/fnins.2025.1677431

**Published:** 2025-10-15

**Authors:** Łukasz Lisowski, Jolanta Lisowska, Anna Charytonowicz, Zofia Mariak, Iwona Obuchowska, Joanna Konopińska

**Affiliations:** ^1^Department of ophthalmology, Medical University of Bialystok, Bialystok, Poland; ^2^Individual Medical Practice Jolanta Lisowska, Bialystok, Poland

**Keywords:** pupil, pupillometry, ocular conditions, pupillary disorders, pupillary reflex, Horner’s syndrome

## Abstract

**Background/objectives:**

The pupil, a dynamic ocular structure, serves as a critical indicator of neurological and ophthalmological function. This interdisciplinary review explores the anatomical, physiological, and pathological aspects of pupillary reflexes and disorders.

**Content:**

Emphasis is placed on the diagnostic relevance of light and accommodation reflexes, sympathetic and parasympathetic pathways, and the clinical implications of conditions such as Horner’s syndrome, Adie’s tonic pupil, and Argyll Robertson pupil. The utility of pharmacological testing and pupillometry in localizing lesions and identifying subclinical changes is extensively discussed. Advancements in pupillary assessment–particularly dynamic and chromatic pupillometry–offer novel insights into autonomic nervous system dysfunction, neurodegenerative diseases, and retinal pathologies including glaucoma and retinitis pigmentosa.

**Recent findings:**

By integrating classical examination techniques with modern imaging and measurement modalities, the pupil emerges as a valuable biomarker in systemic, neurological, and psychiatric disorders. This review underscores the necessity of collaborative, multidisciplinary approaches for accurate diagnosis and effective management of pupillary abnormalities.

**Conclusion:**

Particular attention is given to the diagnostic and prognostic applications of pupillometry, the pathophysiological mechanisms underlying pupillary abnormalities, and the utility of pharmacological tests in lesion localization. By consolidating foundational knowledge with recent clinical and technological developments, this review offers an updated framework for understanding pupillary function and dysfunction and highlights the importance of collaborative approaches across medical specialties for improved diagnostic accuracy and patient care.

## 1 Introduction

The pupil is a natural aperture in the iris that regulates the amount of light entering the eye, reduces chromatic and spherical aberrations on the cornea and lens, mitigates refractive errors by creating a pinhole effect when looking at nearby objects, and increases the depth of field. Under normal conditions, it is round, positioned slightly nasally, and downward from the center. Thus, its center, which is determined by the fovea, may not exactly correspond with the optical axis of the eyeball.

Pupil assessment involves the following: (1) pupillary movements caused by a light stimulus or accommodation, (2) pupil size, (3) pupil symmetry, (4) the effect of pupil size on the optical properties of the eye, and (5) response to medications. The sphincter pupillary muscle is innervated by parasympathetic fibers originating from the cranial nerve III. The dilator pupillary muscle has sympathetic innervation and passes through the superior orbital fissure.

Iris tissue comprises two main layers: the anterior border and posterior epithelial layers. The anterior layer comprises the iris stroma, blood vessels, and nerves supplying the sphincter and dilator pupillary muscles. The posterior layer comprises the dilator and sphincter pupillary muscles and the posterior surface of the pigmented epithelium. The dilator muscle is ring-shaped and located at the iris center. The sphincter muscle is also ring-shaped, located at the iris pupillary margin, and comprises approximately 20 connected motor segments connected with separate innervations through the postganglionic branches of the ciliary nerve. Under normal conditions, these segments simultaneously receive stimuli, and the entire muscle contracts. The normal pupil diameter in adults is reported to range from 3.9 to 4.2 mm (Guillon et al., 2016). Its size changes with age. In newborns, it is narrower than 3 mm. Pupil size increases during the first 6 months of life and reaches its full size in early adulthood. It then decreases again in old age. According to the literature on pupil development, in newborns and infants the diameter is smaller, with an average value of 3.8 ± 0.8 mm (Roarty and Keltner, 1990). This phenomenon is attributed to the immaturity of the nervous system: the pupillary sphincter muscle exhibits a weaker response, and its innervation is still in the process of development. During fetal life, the eyes develop under scotopic conditions. After birth, cones and rods undergo gradual maturation, while the pupillary light reflex–particularly in response to blue light–is mediated by melanopsin-containing retinal ganglion cells (mRGCs) (Ikeda et al., 2015). Consequently, the range of pupillary responses remains limited. This study aimed to highlight the role of pupillary disorders in neurological and ophthalmological diagnoses.

The primary objective of this review is to provide a comprehensive and interdisciplinary synthesis of the current understanding of pupillary disorders, emphasizing their diagnostic significance in ophthalmology, neurology, and systemic medicine. Despite the well-established role of the pupillary light reflex in clinical practice, recent advancements in dynamic pupillometry, chromatic stimulation, and pharmacological testing have expanded the diagnostic utility of the pupil as a noninvasive biomarker for a broad spectrum of diseases. This paper aims to synthesize current diagnostic criteria, highlight recent advances, and provide a clinical guide for practitioners.

In this review, a literature search was conducted in the PubMed database using the keyword “Pupillary light reflex.” The search covered publications from January 1, 1990, to September 2025, without language restrictions. At the initial stage, 548 records were identified, of which 120 articles were selected for full-text review after screening titles and abstracts. Among these, 110 met the inclusion criteria and were incorporated into this review and the bibliography. The manuscript was prepared on the basis of this body of literature.

## 2 Physiological pupillary movements

The pupil is constantly in motion, regardless of changes in the stimuli responsible for its movements, which can be very pronounced or barely noticeable and are considered normal. These should be differentiated from the pathological pupillary symptoms of pinealomas. In pituitary adenomas, these movements are very pronounced and accompanied by other disorders, such as response to light and near-object disruption.

Pupil dilation is a physiological response to skin pricks on the neck and is useful for evaluating potential cranial nerve (CN)-III damage in unconscious patients. This reflex is mediated by the descending branch of the cranial nerve V.

Pupillary abnormalities may include impaired pupillary response to light, abnormal size (narrow, pinpoint pupils or, conversely, dilated pupils), and changes in shape (oval, irregular, or stellate pupils). These abnormalities can develop owing to local causes (within the eye) or systemic diseases (most commonly affecting the nervous system).

### 2.1 Pupillary light reflex

Pupillary light reflex develops as early as the 31st or 32nd gestation week (Alsherbini et al., 2022). In addition to the conducting fibers, the visual pathway also comprises fibers that form part of the reflex-arc afferent pathway, which determines the pupillary light response. This arc comprises three types of fibers: afferent, intraneuronal, and efferent parts. The afferent limb of this reflex is the optic nerve, while the efferent limb is the oculomotor nerve. The retina responds to light stimulation via photoreceptors (cones and rods), bipolar cells, and ganglion cells. The rods are responsible for slight pupillary movements caused by weak light stimuli, whereas cones respond to strong light stimuli. In addition, the retina contains specific melanopsin-containing ganglion cells sensitive to light at 490 nm (blue light). They are responsible for the pupillary light reflex (PLR) and circadian variation in pupil size, which are regulated by the midbrain and hypothalamus, respectively (Hattar et al., 2006; La Morgia et al., 2018).

**FIGURE 1:**
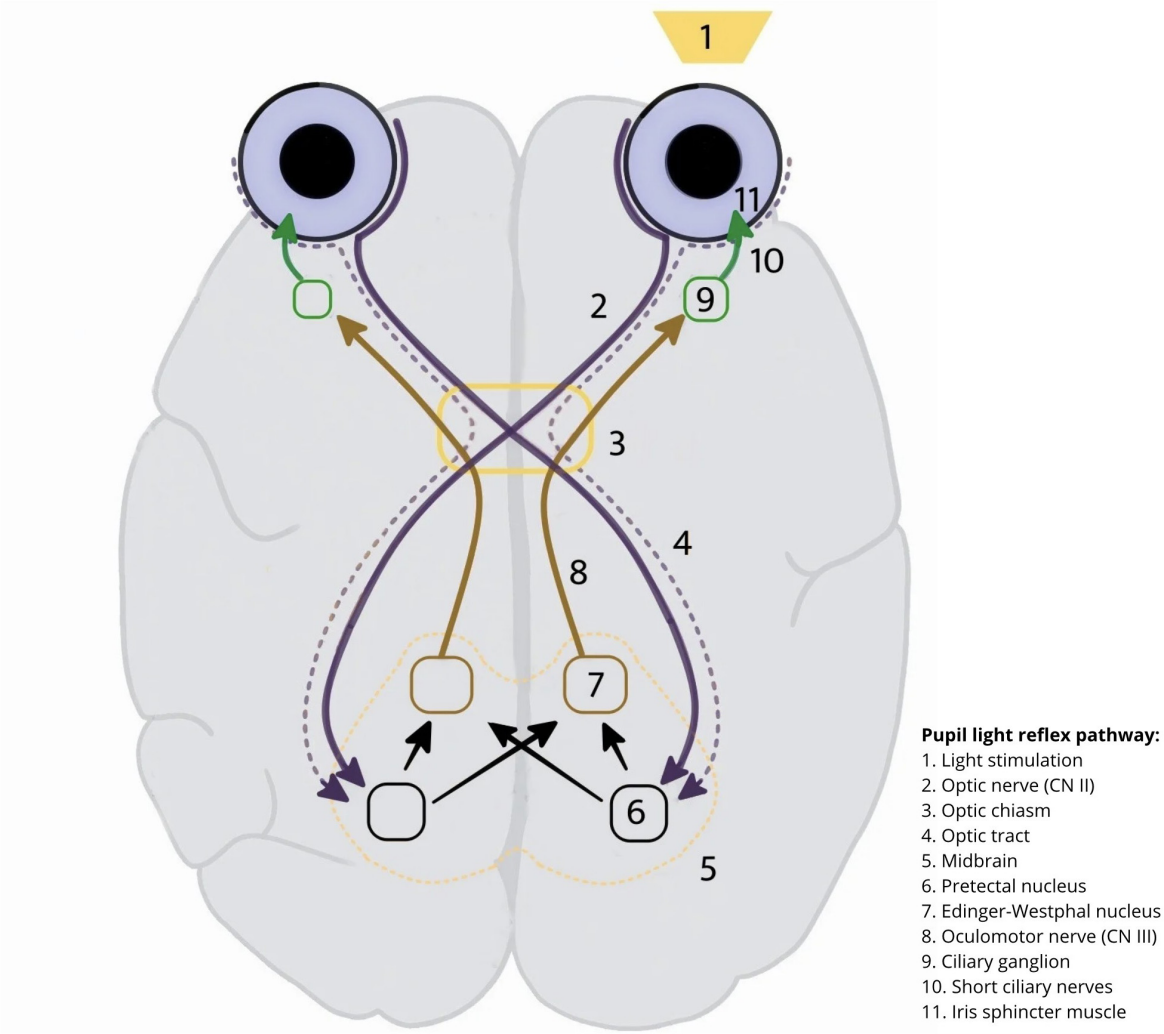
Pupillary light reflex.

The PLR involves four neurons (Figure 1). The first neuron connects the retina to the pretectal nucleus in the midbrain, at the level of the superior colliculus. Stimulation is transmitted along the optic nerve until, together with the fibers that conduct visual sensations, it reaches the lateral geniculate nucleus via the optic chiasm and tract (Tramonti Fantozzi et al., 2021). However, unlike other fibers, they do not break off but continue toward the tectum, more precisely toward the superior colliculus, and terminate at the pretectal region on the border between the midbrain and the diencephalon. Visual nerve impulses from the nasal retina travel through nerve fibers that cross the chiasma opticum to reach the opposite pretectal nuclei. Impulses originating from the temporal retina reach the pretectal nucleus on the same side. They pass from the cells in this stimulation region to the nuclei of CN III, the second neuron involved in this reflex, connecting the pretectal nucleus with both Edinger–Westphal nuclei on the same and opposite sides. Consequently, when one eye is exposed to light, both pupils contract symmetrically. The third neuron connects the Edinger–Westphal nucleus to the ciliary ganglion via the oculomotor nerve. The preganglionic nerve fibers travel to the ciliary ganglion, where the postganglionic nerve fibers (via the short ciliary nerves) reach the sphincter pupillary muscle, thus forming the fourth neuron involved in this reflex. The entire reflex arc is outlined as follows: optic nerve → optic tract → superior geniculate nucleus (pretectal area) → Edinger–Westphal nucleus → preganglionic nerve fibers (CN III) → ciliary ganglion → postganglionic nerve fibers (short ciliary nerves) → sphincter pupillae muscle.

The stimuli transmitted from one optic nerve reach both sides of the brainstem. Under normal conditions, a light stimulus in one eye constricts the pupils of the other (a consensual response) because the stimulation of one optic nerve affects both Edinger–Westphal nuclei and passes through the parasympathetic nerve fibers, constricting the pupils of both oculomotor nerves (Belliveau et al., 2025; Young and Lund, 1994). In most people, the number of uncrossed and crossed fibers is equal, preventing anisocoria when one eye is exposed to light. Crossed fibers are more numerous than uncrossed fibers in a small part of the population, indicating that when only one eye is exposed to light, the reaction of the other eye is slightly less noticeable, which is normal in some mammals (e.g., cats). Rabbits or birds with fully crossed fibers have no consensual response.

The absence of a direct PLR in the stimulated eye may indicate optic or oculomotor nerve damage. Regarding optic nerve damage, shining a light into the affected eye will not cause pupil size changes in either eye, whereas shining a light into the healthy eye will cause both pupils to constrict (Lee and Lee, 2025; Yoo et al., 2017). Conversely, in oculomotor nerve damage, shining a light into the affected eye causes the opposite pupil to constrict, while the affected pupil shows no direct or consensual response (Kim et al., 2018; Larsson, 2015; Lin and Hu, 2019).

The ophthalmic branch of the trigeminal nerve also plays a minor role in sphincter pupillae innervation. However, sensory nerves may contribute to regulating pupil size. Generally, iris stimulation during cataract surgery may cause the pupil to contract despite pharmacologically induced mydriasis (Möller et al., 2000). The pupillary reflex can occur in a person who has no visual function after damage to the higher levels of the visual pathway. This is because the pupillary fibers separate from the visual fibers going to the visual cortex at the level of the lateral corpus callosum (Kelbsch et al., 2022).

## 3 PLR abnormalities

Damage to PLR afferent pathways may cause abnormalities in the pupillary response to light, depending on the severity and location. The most severe abnormality is the absence of light stimuli perception (practical blindness), triggered by optic nerve damage (due to injury) or retinal damage, where there is a complete lack of the PLR when the eye on the affected side is exposed to light (amaurotic pupil) (Hall and Chilcott, 2018). It is caused by a completely damaged CN II. The eyes on the affected side do not perceive light, and the pupils are equal in size. When the affected eye is stimulated by light, it does not respond, unlike the healthy eye. The pupillary response to a direct light stimulus in the other eye remains intact, with no consensual response to the light stimulus in the amaurotic pupil. The accommodation reflex is also preserved. Lack of consensual response implies that both (afferent and efferent) reflex arc pathways are impaired, indicating that CN II and III are damaged, as observed in conditions secondary to orbital apex syndrome (Aguirre and Nene, 2025; Siva Kumar et al., 2021).

For severe visual pathway damage (total damage to the optic tract), the pupil on the affected side reacts only when the nasal retina is exposed to light (Wernicke’s pupil) (Xu et al., 2024). Conversely, the healthy eye elicits responses only when light stimuli are applied to the temporal retina. In such cases, the pupils are equal in size, and accommodation reflex is preserved, accompanied by hemianopia in both eyes (Chen et al., 2011).

Regarding the Marcus Gunn pupil, a weakened PLR occurs on the affected side, along with a paradoxical pupillary dilation to alternating light stimulation (where the pupil dilates on the affected side of the visual pathway in the brain instead of constricting when exposed to light), which could also be the case in partial optic nerve damage and severe retinal disease (Simakurthy and Tripathy, 2025).

In methanol-induced optic neuropathy, the PLR is weakened or absent often in both eyes, accompanied by a rapid decline in the visual acuity of both eyes as well as xanthopsia and large scotomas in the field of vision. Over time, visual acuity severely declines because of toxic optic atrophy (Liberski et al., 2022; Sun et al., 2022).

Hutchinson’s pupil is a dilated, fixed pupil that responds to increased intracranial pressure caused by an ipsilateral lesion (subdural hematoma or CN III compression by the dorsum sellae) or bilaterally when pressure on the tentorium cerebelli causes brain compression (McAvoy et al., 2022; Pearce, 2018).

### 3.1 Accommodation reflex

Accommodation reflex is an optic nerve reflex that is triggered when looking at near objects; the ciliary muscles contract, and the lens becomes more convex. Concurrently, the eyes move toward each other (convergence), and both pupils constrict. This triad of symptoms is called the near response. These reactions are co-movements because they can occur independently. The afferent part of this reflex arc starts in the retina and travels through the optic nerve, chiasm, and tract to the lateral geniculate nucleus and onto the occipital cortex. Subsequently, the stimulation travels through the tractus occipitomesencephalicus to the midbrain, nucleus of Perlia, and Edinger–Westphal nuclei. Further, the course of the efferent fibers through CN III is similar to the efferent fibers for the light reflexes, while the responses differ in the afferent pathway. This reflex may be absent despite PLR preservation. Conversely, the absence of PLR may be accompanied by a normal accommodation reflex (Argyll Robertson sign) (Wu et al., 2022). The postganglionic parasympathetic accommodative axons that innervate the ciliary body smooth muscles outnumber the light-reflex axons that innervate the sphincter pupillae in a ratio of 30:1. In summary, when looking at near objects, accommodative neurons (which cause ciliary muscle contraction) and photoreceptors (which cause sphincter pupillary contraction) in the Edinger–Westphal nuclei are stimulated at the supranuclear level, triggering separate responses in the accommodation and light reflexes, which are carried along the oculomotor nerve to the ciliary ganglion, with impulses to the ciliary muscles and sphincter pupillae. The preganglionic and ganglionic pathways involved in light-induced pupillary constriction are the same as those activated when looking at nearby objects (Motlagh and Geetha, 2025).

### 3.2 Light-near dissociation

Pupils respond equally to light and near objects. If one of these reflexes dominates, the condition is referred to as light-near dissociation, which results from three mechanisms:

- Loss of light sensitivity due to severe afferent system damage (the retina, optic nerve, and optic chiasm).
- Disruption of light reflex conduction to the Edinger–Westphal nucleus from the pretectal area, where damage occurs in the midbrain tectum (secondary to infection, Argyll Robertson pupil, pineal region tumor, or stroke [dorsal midbrain syndrome]).
- Aberrant reinnervation of the sphincter pupillae by accommodative fibers (Adie’s syndrome) or extraocular muscle fibers of the oculomotor nerve (medial rectus or accommodative fibers of CN III).

The disassociation may be unilateral, caused by afferent pathway damage (herpes zoster ophthalmicus, CN III aberrations), or bilateral secondary to early-onset diabetes, myotonic dystrophy, dorsal midbrain syndrome, Argyll Robertson pupils, pituitary tumors, amyloidosis, encephalitis, or chronic alcoholism (Kawasaki, 2014; Shams et al., 2010).

### 3.3 Pupillary dilation reflex

Pupillary dilation is mediated by sympathetic, central, and peripheral nerve fibers (Manjunathan and Saini, 2022). The pupillary dilation reflex involves three pairs of neurons that do not cross the right and left sides of the body. The first neuron originates from the posterior hypothalamus and descends through the brainstem on either side of the lateral parts of the medulla oblongata to the ciliospinal center, located at the cervicothoracic segment C8–Th2. The second neuron is formed by the sympathetic fibers of the ciliospinal center that travel to the superior cervical ganglion. Preganglionic fibers leave the spinal cord level and pass through the apex of the pleura, lung, and vertebral arms, branching off to both sides of the neck in the superior cervical ganglion at the carotid bifurcation level. The third (postganglionic) neuron runs along the internal carotid artery to the skull foramen, joins the ophthalmic branch of the trigeminal nerve, and enters the ciliary body and the dilator pupillae along with the nasociliary and ciliary nerves. It connects to the abducens and trigeminal nerves before entering the eye socket and innervating the dilator pupillae via the long ciliary nerves (Mombaerts, 2025).

Efferent fibers from the frontal lobes, located in the dorsolateral part of the medulla oblongata, run through the brainstem; in their further course in the cervical spinal cord, they pass through the lateral cords and terminate in the ciliospinal center, which is located in the C8 and T1 segments of the spinal cord (Wilhelm, 2011).

From the sympathetic center, preganglionic fibers (rami communicans albi) enter the cervical ganglia via the nerve roots. In the superior cervical ganglion, these fibers switch. Postganglionic fibers enter the cranial cavity along the internal carotid artery (via the foramen lacerum medium). In the cavernous sinus, these fibers form part of the carotid artery plexus, entering the orbit through the sphenopetrosal fissure as long ciliary nerves; some of which run to the dilator pupillae.

Under normal conditions, pupil dilation involves two processes: sphincter pupillae relaxation and dilator pupillae contraction. Because the sphincter pupillae is stronger than the dilator pupillae, the pupil does not dilate easily until the sphincter relaxes. The sphincter pupillae muscle is relaxed by the supranuclear inhibition of the Edinger–Westphal nucleus in the brainstem, which is achieved by sympathetic alpha-2 receptor activation in neurons passing through the periaqueductal gray matter and reaching the efferent fibers of neurons in these nuclei. Activating this central inhibition also inhibits the parasympathetic pathway of pupillary contraction, resulting in pupillary dilation. During sleep, general anesthesia, or under narcotic influence, supranuclear inhibition is inactive, which manifests as permanent pupillary constriction. Edinger–Westphal neurons are unique in this regard. Their resting potential is very high; therefore, they frequently discharge without any inhibition, causing sustained pupillary contractions. However, during wakefulness, inhibition recurs, causing pupillary dilation. The light stimulus projected onto the retina reaches the Edinger–Westphal nucleus, which overrides this braking mechanism, causing another pupillary contraction.

Peripheral sympathetic nervous system stimulation also affects pupil size. Its activation increases pupillary constriction dynamics and maximum pupil size but is not necessary to initiate constriction. Peripherally circulating catecholamines can affect dilator pupillae movement directly through blood and indirectly through tears, causing mydriasis (Wilhelm et al., 2007).

The balance among these three factors affecting pupil size is essential, and its disruption can result in pupil size, shape, and symmetry abnormalities.

Pupil dilation involves two mechanisms:

First, when light no longer stimulates the retina, the Edinger–Westphal nuclei in the midbrain are inhibited, reducing impulses from the preganglionic parasympathetic neurons in the Edinger–Westphal nuclei, thereby causing sphincter relaxation. Second, the sympathetic stimulation increases in seconds, causing the dilator pupillary muscle to actively contract and dilate the pupil. Both reflexes are closely related.

Abnormal pupillary dilatation can be caused by the following: mechanical restrictions of the sphincter pupillae (scarring caused by infection or surgery or acute trauma triggering prostaglandin release), drug-induced miosis (effect of cholinergic drugs), aberrant reinnervation of the sphincter pupillae by cholinergic neurons (usually inhibited under low-light conditions [accommodative neurons or extraocular muscles, chronic Adie’s syndrome]), lack of inhibitory signal arising in the Edinger–Westphal nuclei (fatigue, drowsiness, general anesthesia, narcotics), damage to inhibitory fibers in the periaqueductal gray matter (secondary to inflammation, infection, and lymphoma), old age (bilateral miosis), absence of sympathetic dilator pupillae stimulation, and sympathetic neuron damage (oculosympathetic defect in Horner’s syndrome).

Pupils in dorsal midbrain syndrome (Parinaud’s syndrome) typically present with bilateral dilation of the pupils, which do not constrict or only slightly constrict in response to light but constrict when looking at near objects (light-near dissociation in the tectum), which may be accompanied by other ocular symptoms, such as eyelid retraction, vertical gaze palsy, convergence retraction nystagmus, upward strabismus, and torsional nystagmus (Ortiz et al., 2021). This dissociation originates in the dorsal (tectal) part of the midbrain. In contrast, lesions involving the ventral midbrain result in pupils that also fail to constrict during near focus. This disorder is caused by centripetal pathway disconnection of the pupillary reflex in the metatrochlear region from the central parasympathetic nucleus (Edinger–Westphal). It can be caused by hydrocephalus, a tumor in the pineal gland or midbrain cover, a demyelinating process, or stroke. A magnetic resonance imaging scan of the head may reveal ventricle enlargements or signal disturbances in the pineal gland or midbrain (Ortiz et al., 2021).

### 3.4 Argyll robertson pupil

**FIGURE 2:**
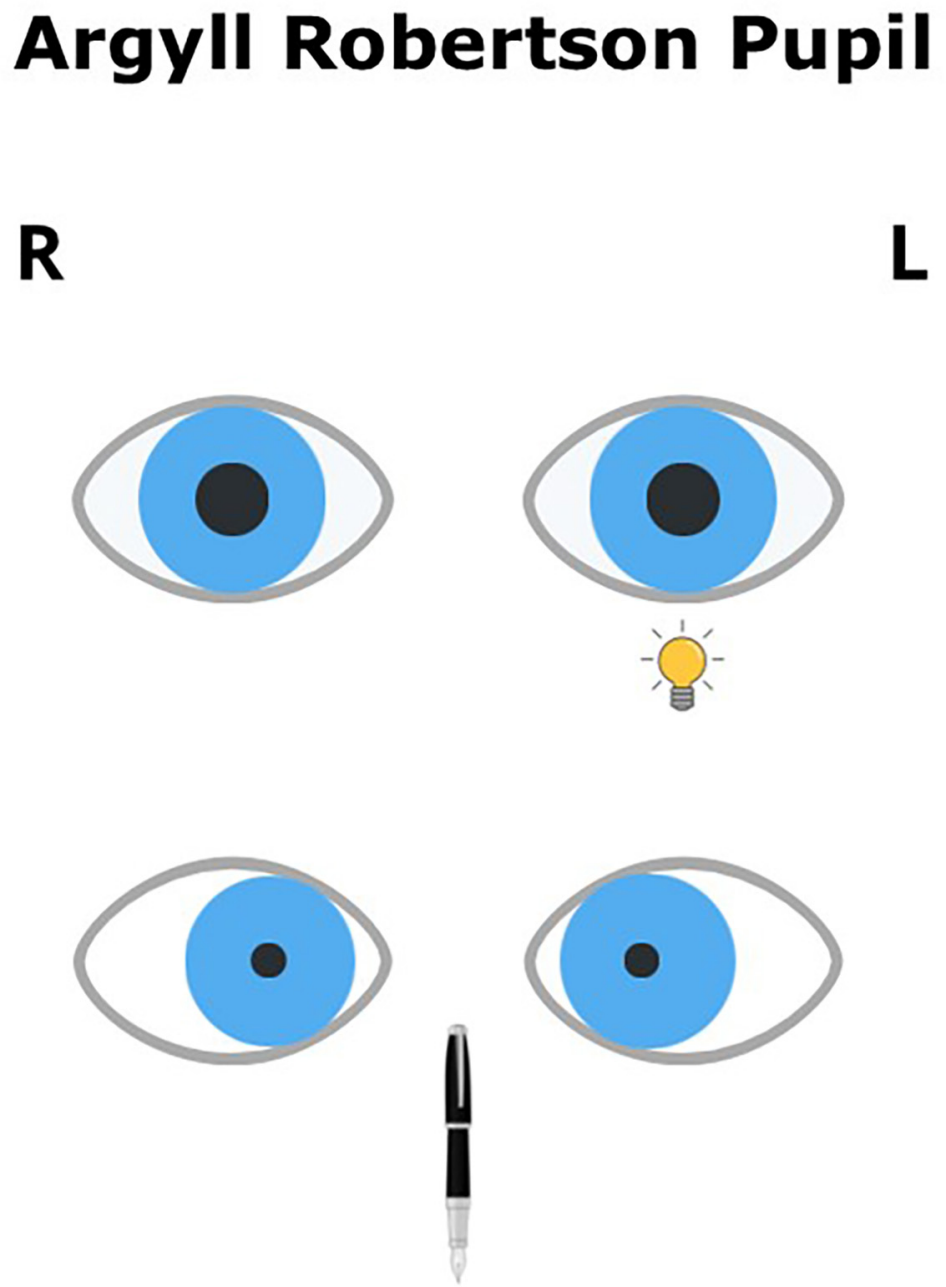
Argyll Robertson pupil.

First described in 1869, Argyll Robertson pupil refers to a condition in which the pupils exhibit preserved convergence and accommodation responses (although not entirely normal), while the direct and consensual light reflexes are absent. Both pupils are narrow (<3 mm), irregular, and often decentered (Figure 2). The pupils respond poorly to mydriases.

Argyll Robertson pupil is caused by damage in the Sylvian aqueduct area, where fibers that form the afferent part of the reflex arc are located, responsible for the PLR. It is usually secondary to neurosyphilis, herpes zoster infection, encephalitis, or syringobulbia. Other less common causes include multiple sclerosis, collagenosis, diabetes, neurodegenerative diseases or chronic alcoholism. In addition, it may result from a mass in the Sylvian aqueduct area, located between the third and fourth ventricles, or occur secondary to general paresis. The fibers that conduct the accommodation reflex have a different course and are thus preserved in the early stages of the disease. As the disease (spinal atrophy or general paresis) progresses, damage to fibers may also occur, leading to complete light and convergence reflex abolition (rigid, very narrow, and pinpoint pupils) (Thompson and Kardon, 2006).

## 4 Pupillary reflex testing

### 4.1 Light reflex test

Light reflex test can be performed in daylight or under electric light, but the lighting should be dimmed, with just enough illumination to visualize the iris and pupil. The patient sits facing the light. The person conducting the test covers both eyes with their hands and asks the patient to keep their eyes open and look at a distance. Each eye is assessed separately. With quick movement, one eye is uncovered (the other remains covered), and pupil behavior is observed. Pupils will constrict under physiological conditions. After examining one eye, the other eye is examined in the same manner. For electric light, a quick movement is used to cast a beam of light on the pupil of one eye, and subsequently on the other. Pupils constrict following exposure to light. During the examination, the patient looks at a distance to avoid accommodation-induced pupil constriction reactions (Belliveau et al., 2025).

The consensual reflex test involves observing the indirect reflex (i.e., the behavior of the pupil opposite to that exposed to light) (Spector, 1990).

The accommodation reflex test involves asking the patient to look at a distance and then quickly look at an object (finger) located just in front of the eyes (6–7 cm). Under normal conditions, the pupils clearly constrict when looking at a nearby object. When looking at a nearby object, the eyeballs also move toward each other (converge). Thus, the accommodation reflex is tested simultaneously with the convergence reflex (Motlagh and Geetha, 2025).

**FIGURE 3:**
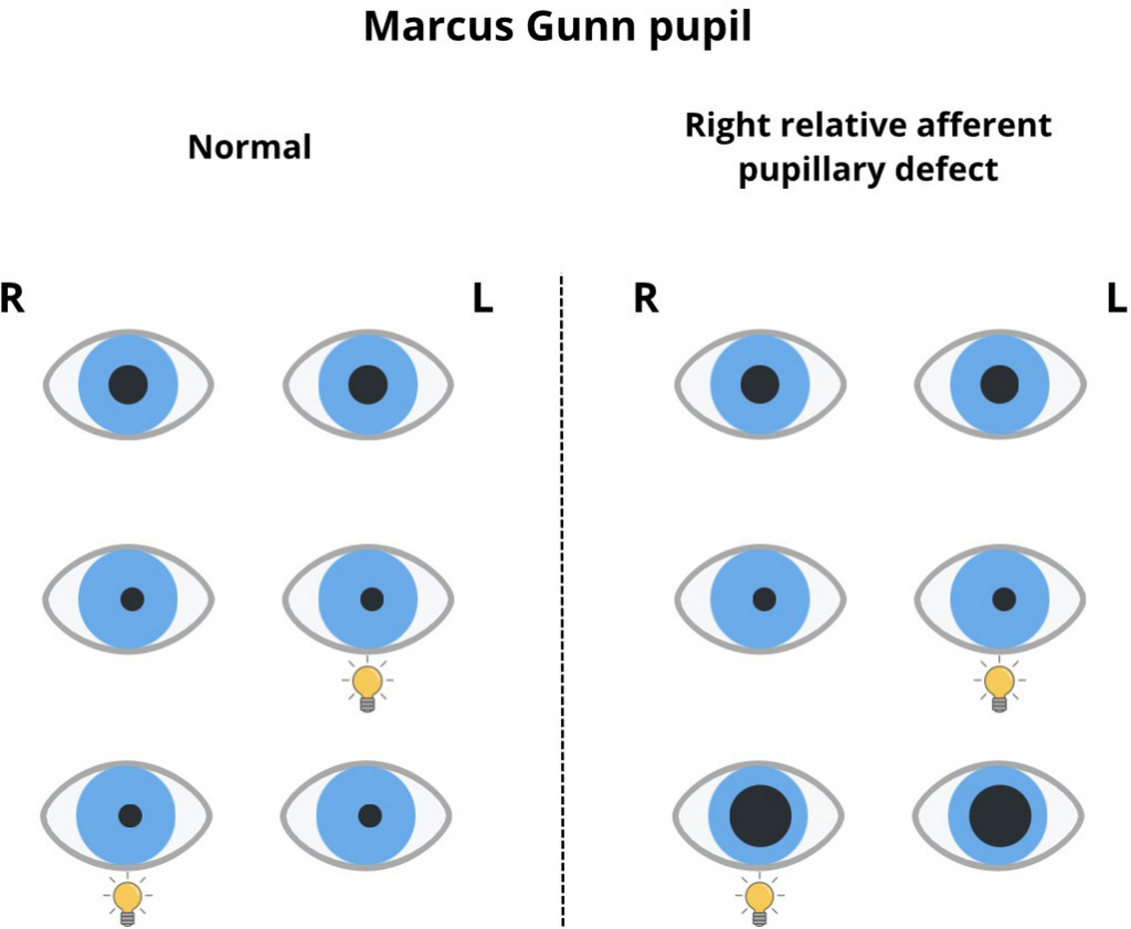
Marcus Gunn pupil (right relative afferent pupillary defect).

The relative afferent pupillary defect (the Marcus Gunn pupil) is dependent on the reduced reaction to light that occurs with a defect in conduction in one optic nerve (Sharifi et al., 2021). It is caused by asymmetrical nerve impulse conduction in both optic nerves. Clinically, it is characterized by a weakened pupillary reaction to light in the affected eye and a paradoxical pupillary reaction in the “alternating light stimulation test” (Figure 3). To trigger this, a light source is used to illuminate each eye alternately at very short intervals. The room lighting must be dim, and the patient must focus on a distant object. Both pupils react even though the conductivity in one optic nerve is impaired. When the light source is moved quickly from a healthy pupil to a pupil with an afferent defect, both pupils dilate. The brain interprets a decrease in stimulation conducted by the nerve with the defect as a decrease in light source intensity. Another theory explaining this symptom states that pupil dilation caused by the removal of light from the healthy eye outweighs the constriction caused by light stimulation of the affected eye, which occurs in optic nerve diseases (retrobulbar neuritis) or optic chiasm pathologies if one of the nerves is affected. It can also occur in serious retinal diseases, such as diabetic proliferative retinopathy with total retinal involvement, retinal detachment, or significant amblyopia. In contrast, changes in optical centers, such as mature cataracts, do not weaken the centripetal light-reflex pathway (Broadway, 2012).

### 4.2 Pupillometry

The PLR can also be used to obtain objective information regarding the sensitivity of small areas of the visual field to light by tracking small pupillary constrictions in response to local stimulation at different locations in the visual field. Automatic perimetry can be adapted to record such pupillary responses to each local light stimulation to recreate an objective map of these reactions. A video camera is directed at the pupil, and the amplitude of each contraction is measured and recorded using a computer, allowing the localization of defects in the pathways involved in pupil contraction. This test is also useful for non-organic, functional visual field defects when the patient simulates a lack of light stimuli visibility. A light stimulus that causes pupillary contraction triggers this reaction across the entire retinal surface. Therefore, damage to a part of the retina can affect the size, speed, and contraction strength. The loss of only 5° of the visual field can reduce pupil reactivity to light. In addition, the loss of the temporal visual field results in greater light reflex impairment than the loss of the nasal visual field (Kardon et al., 2006; Kawasaki, 2014).

In recent years, several modern methods and devices for assessing the PLR have been developed. Gray et al. (2024) demonstrated that portable, handheld pupillometers (e.g., NeuroLight) enable reliable PLR measurements under field conditions, although latency assessments were less precise when using a smartphone. Ben Barak-Dror et al. (2024) introduced a touchless short-wave infrared system for PLR monitoring, allowing dynamic recording of pupillary responses even with closed eyelids. Similarly, Temel et al. (2019) developed a head-mounted device for automated RAPD assessment, employing IR cameras and RGB diodes. The development of these technologies highlights the growing importance of mobile, contactless, and automated pupillometry in ophthalmic and neurological diagnostics, particularly in field settings and intensive care.

Pupillometry analyzes the dynamics of pupil size changes and is a key diagnostic tool in ophthalmology, neurology, and psychophysiology. PLR provides valuable information about the functioning of the autonomic nervous system and sensory integration within the visual system. This reflex is an adaptive mechanism that regulates the quantity of light reaching the retina, protecting it from overexposure and simultaneously providing optimal vision conditions (Mathôt, 2018).

Pupillometry is widely used in neuro-ophthalmology, not only as a method of measuring pupil size but also as a tool for the quantitative assessment of the relative afferent pupillary defect, field of vision estimation, assessment of optic nerve function (such as in glaucoma neuropathy and optic neuritis accompanying multiple sclerosis or myasthenia gravis), and retinal function analysis in diseases such as retinitis pigmentosa, central retinal vein occlusion, or age-related macular degeneration (Hall and Chilcott, 2018; Rukmini et al., 2019). Recently, pupillometry has also been used in monitoring patients in intensive care units, especially in assessing pupillary responses as a non-invasive indicator of increased intracranial pressure in patients with severe brain injuries and in assessing response to analgesic treatment (Chen et al., 2011; Jahns et al., 2019; Lukaszewicz et al., 2015). Moreover, It is also increasingly used in psychiatry to support affective and anxiety disorder diagnoses (Fietz et al., 2024; Thakkar et al., 2018) and in research on neurodegenerative diseases of the central nervous system, such as Parkinson’s (PD) and Alzheimer’s diseases (17).

Pupillary activity can be divided into two basic types: the PLR and spontaneous pupillary activity (hippus). The PLR is the dynamic response of the pupil to light stimuli. After a period of latency (the time between exposure to light and the reaction onset), the pupil constricts at a certain speed to reach its minimum diameter, after which it expands again (Steiner and de Zeeuw, 2024).

**FIGURE 4:**
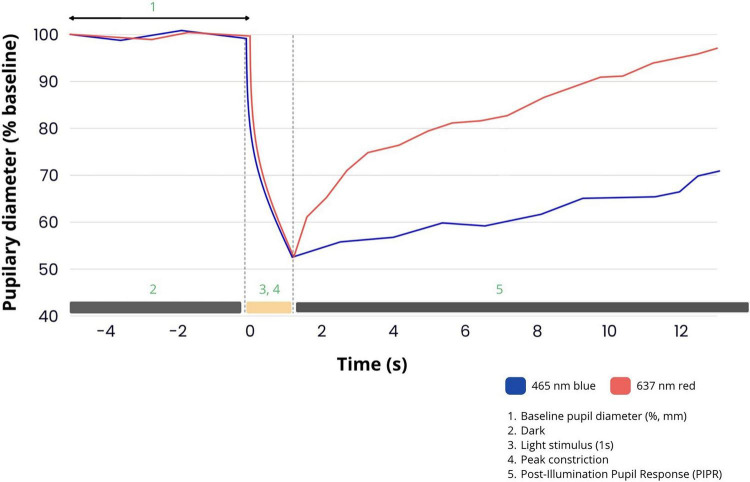
Pupillometric response to blue and red light stimuli.

When assessing PLR, several quantitative parameters are analyzed, including pupil diameter before stimulation (baseline) and after maximum contraction (minimum), response amplitude and relative amplitude, latency, time to reach maximum contraction, maximum contraction velocity and acceleration, diastole duration, maximum diastolic velocity, and post-illumination pupillary response (PIPR). These tests can include both single and repeated light stimulations with defined characteristics such as intensity (10 or 100 cd/m^2^), wavelength (white and red light of 635 nm and blue light of 470 nm), pulse duration, and flash frequency (Figure 4). The choice of light stimulation parameters determines which photoreceptor populations will be activated: either rods that react to light via rhodopsin, cones that use different opsins, or intrinsically photosensitive retinal ganglion cells (ipRGC) whose activity depends on the presence of melanopsin. Measurements are taken in the monocular or binocular mode, which allows the recording of both direct and indirect pupillary reflexes. The pupillary response is also influenced by several external and physiological factors, including background luminance during stimulation and the time required for the patient to adapt to both dark and light conditions. Proper experimental condition preparation, including light background standardization and ensuring sufficient adaptation time, is crucial for obtaining repeatable and reliable pupillometric results (Kelbsch et al., 2019).

The second type of recorded pupillary activity, the hippus, is characterized by spontaneous, rhythmic pupil diameter oscillations. Their frequency depends, among other factors, on heart and respiratory rates. The analysis of the hippus considers the amplitude, frequency, and maximum speed of diameter changes (Bouma and Baghuis, 1971; Turnbull et al., 2017).

The PLR results from a complex interaction between retina photoreceptors and integrative structures in the central nervous system that coordinate iris muscle activity. The human retina has three main photoreceptor types: rods, cones, and melanopsin-containing ganglion cells (ipRGCs). Rods (dominant in the peripheral part of the retina) are extremely light-sensitive and enable vision under low-light conditions. The absorption spectrum corresponds to a maximum wavelength of approximately 498 nm. Cones, however, are responsible for color vision and function in bright light, with three subtypes: S cones, sensitive to short wavelengths (approximately 420 nm); M cones, responsible for light perception with medium wavelengths (approximately 530 nm); and L cones, which react to light with long wavelengths (approximately 560 nm) (Park et al., 2011; Rukmini et al., 2019). The rods and cones transmit information through a layer of bipolar cells to the ganglion cells, which send the signal to higher levels of the visual system (Masland, 2012).

Recently, interest has been growing in the use of pupillometry in research on ipRGC functions. These cells account for approximately 0.2% of all retinal ganglion cells and have the unique ability to react to light independently because of the presence of melanopsin, whose absorption spectrum peaks at 482 nm (Dacey et al., 2005; Provencio et al., 2000). In response to blue light stimuli, high pupil contraction amplitudes are recorded, compared with reactions caused by red light. Unlike rods and cones, ipRGCs can be stimulated for a long time, making them key regulators of the pupillary reflex and circadian rhythm (Markwell et al., 2010; McDougal and Gamlin, 2010). Their activity can be studied by analyzing the PIPR, which is the persistent contraction of the pupil after the end of light exposure (La Morgia et al., 2018). This phenomenon has also been confirmed in studies on laboratory animals without active rods and cones, where pupillary responses to blue light are still observed despite no classical photophobia. Furthermore, these animals show preserved circadian rhythms of sleep and wakefulness, suggesting that melanopsin-containing ganglion cells are crucial not only in the pupillary reflex but also in circadian rhythm regulation (Barnard et al., 2004).

The function of individual photosensitive cell populations can be evaluated by selecting appropriate pupillographic parameters.

Pupillometry is a particularly sensitive and noninvasive method for assessing autonomic nervous system function. Pupil size, dynamics, and response to light stimuli are important indicators of nervous system integrity. The autonomic nervous system is essential for regulating PLR. The parasympathetic and sympathetic pathways are responsible for pupil contraction (myosis) and dilation (mydriasis), respectively (Bremner and Smith, 2006; Tsitsi et al., 2023).

The maximum heart rate is primarily triggered by light-intensity changes; however, its course can also be modulated by cognitive factors, stress, medications, and various neurological and systemic diseases (Ciuffreda et al., 2017).

A reduction in the initial pupil diameter and reflex amplitude weakening occur in the subclinical phase of diabetic autonomic neuropathy, even before eye fundus changes characteristic of diabetic retinopathy develop (Bista Karki et al., 2020; Cankurtaran et al., 2019; Erdem et al., 2020; Karavanaki et al., 1994).

Similarly, early pupillary disorders resulting from cholinergic deficits have been observed in patients with PD and Alzheimer’s diseases. In PD, pupillometry reveals several characteristic abnormalities reflecting autonomic nervous system dysfunction. A frequently observed abnormality is reduced speed and maximum pupil contraction acceleration (Fotiou et al., 2009; Stergiou et al., 2009). These parameters can change even when other symptoms remain scarce. Occasionally, a reduced initial pupil diameter in darkness (resting myosis) is also observed, which may be due to an imbalance between the sympathetic and parasympathetic components of the autonomic nervous system (Tsitsi et al., 2023). In addition, recent reports describe abnormalities in the hippus– the rhythmic pupil diameter oscillations–which may be weakened or irregular in patients with PD, also indicating autonomic system dysregulation (Mangipudi et al., 2015). Dynamic pupillometry can provide useful diagnostic information and support disease progression or treatment response monitoring.

In glaucoma diagnosis, a new technique termed “pupillary perimetry” is currently being developed for assessing retinal function asymmetry by analyzing pupillary amplitude in response to light stimuli applied in different quadrants of the visual field. The correlation between the results and classical perimetry is approximately 84% and increases with change severity (Carle et al., 2014; Maddess et al., 2009).

In glaucoma, progressive damage occurs to retinal ganglion cells, including the ipRGC population. Their dysfunction is reflected in PIPR analysis. Under physiological conditions, ipRGCs show prolonged activation after blue light stimulation, which manifests as prolonged pupil constriction. In patients with glaucoma, this response is markedly weakened and shortened, suggesting impaired ipRGC function. Therefore, PIPR measurement using blue light is a potentially sensitive biomarker for glaucoma changes–especially useful for detecting subclinical retinal dysfunction in diagnostically difficult cases or early disease stages (Feigl et al., 2011; Kelbsch et al., 2016).

Other retinal diseases, such as retinitis pigmentosa (RP), also exhibit characteristic changes in the pupillary response to light. RP, a progressive degenerative disease leading to gradual rod and cone function loss, causes classic PLR weakening. Pupil contraction amplitude is reduced and may completely disappear in advanced stages, especially when stimulated with red light, which mainly activates the cones. Despite this, some patients maintain a response to blue light, which is related to the activity of ipRGCs that are relatively resistant to degeneration in RP. Consequently, recording the PIPR, which is more pronounced after blue light stimulation than after red light stimulation, becomes possible. In many cases, the pupil returns to its original diameter more slowly after blue light exposure, suggesting a disinhibited response from ipRGCs (Kardon et al., 2011; Kelbsch et al., 2017).

Notably, even in patients with a completely extinguished electroretinographic response, obtaining a pupillary response in pupillometry is possible, making this method potentially useful for assessing residual retinal function, which is particularly important for qualifying experimental therapies such as gene therapies or retinal prosthesis implantation, as it indicates preserved retinal nerve cell activity despite considerable photoreceptor loss (Lisowska et al., 2017).

In a study by Jaworski et al. (2025) the authors presented a comparison between an AI-based mobile pupillometry system (AI Pupillometer) with the NPi-200 device for measuring PLR and exploring its correlation with glaucoma-related markers. The study comprised two main experiments: in Experiment 1, 20 healthy participants underwent PLR assessments using both the AI Pupillometer and the NPi-200 device. Experiment 2 involved 46 participants, including 24 with primary open-angle glaucoma (POAG) and 22 healthy controls. PLR measurements from the AI Pupillometer were correlated with structural and functional ocular parameters. An additional study expanded the sample to 387 participants (103 glaucoma patients, 284 controls), focusing on differential pupillometry parameters to minimize ambient light interference. The results of Experiment 1 showed strong correlations between the AI Pupillometer and NPi-200 in key parameters such as initial pupil size, constricted pupil size, and constriction velocity, confirming the AI Pupillometer’s reliability. In Experiment 2, while no statistically significant differences in light-corrected PLR parameters were found between groups, patients with glaucoma had a marginally higher constricted pupil size. Significant correlations were observed between pupillometry and advanced ocular imaging results, notably between constriction amplitude and visual field loss. The additional study revealed significant differences in constriction amplitude and relative pupil size change between glaucoma patients and controls, reinforcing the AI Pupillometer’s diagnostic potential. The research also uncovered previously unexplored relationships between PLR and parameters, such as retinal vessel density and gaze fixation stability. These findings suggest that mobile PLR technology could play a significant role in expanding glaucoma screening capabilities, particularly in areas with limited access to specialized ophthalmic equipment.

Go et al. (2020) conducted experiments to quantify the relationship between PLR parameters and illumination levels. They developed lighting-invariant versions of these parameters and demonstrated their ability to discriminate between reactive and unreactive pupils. A smartphone app was used to measure PLR, and tropicamide eye drops were administered to simulate unreactive pupils. The study resulted in the creation of the Pupil Reactivity (PuRe) score, a lighting-corrected index that quantifies pupil reactivity on a scale of 0–5. This score was shown to be stable across various lighting conditions while maintaining sensitivity to pupil reactivity changes. The researchers argue that this lighting-invariant quantitative pupillometry could improve the reliability and accessibility of PLR measurements in clinical settings, potentially enhancing neurological monitoring and decision-making in critical care.

A different study (Yoshida and Kawabata, 2018) assessed PLR in patients with delirium following traumatic brain injury (TBI), using a pupillometer to enable early detection and potential prevention of delirium. The authors investigated the correlation between the pupillary PLR, measured with a pupillometer during mechanical ventilation, and the development of postextubation delirium. Pupillometry was performed in the first 3 days after admission to the intensive care unit. Ten patients with TBI (38%) were diagnosed with postextubation delirium, whereas 16 (62%) were not. Significant correlations between delirium and 2 PLR variables were found: pupil constriction percentage [*r*_*pb*_(24) = −0.526, *P* = 0.006] and constriction velocity [*r*_*pb*_(24) = −0.485, *P* = 0.012]. The *t* test also revealed a significant difference in constriction percentage and velocity scores between patients with TBI with and without delirium (*P* ≤ 0.01). This study suggests that the use of a pupillometer may help identify patients with brain injury at risk for delirium.

Uludağ et al. (2017) developed a machine learning approach for ambient-light-corrected parameters and the PuRe score in smartphone-based pupillometry. The research addressed the challenge of accurately measuring the PLR under varying lighting conditions. First, the researchers quantified the effect of ambient light on various PLR parameters using a smartphone-based pupillometer. Subsequently, they developed lighting-invariant versions of these parameters and demonstrated that these corrected parameters could still effectively discriminate between reactive and unreactive pupils. The team also created a composite PuRe score that optimally distinguishes between reactive and unreactive pupils while remaining stable across different lighting conditions. They introduced a novel approach to correct for ambient light effects on pupillometry, which has been a significant challenge in clinical settings. Additionally, they demonstrated the possibility to obtain reliable PLR measurements across a wide range of lighting conditions using a standard smartphone, potentially expanding the accessibility of quantitative pupillometry in various conditions and opening up new possibilities for neurological monitoring in diverse settings.

Additionally, the PLR test can be helpful in differentiating the causes of anisocoria. It enables the rapid distinction between physiological and pathological changes, the identification of potential parasympathetic or sympathetic pathway involvement, and, when necessary, the prompt referral of the patient for further urgent diagnostics. This examination is particularly useful in distinguishing physiological anisocoria (Kılınç Hekimsoy et al., 2022), which is characterized by normal pupillary responses to light and accommodation, from Horner’s syndrome, in which the difference in pupil size becomes more pronounced in darkness due to impaired dilation of the affected pupil (Disse et al., 2025). Thus, the PLR represents an important tool in everyday clinical practice for the rapid and accurate assessment of the nature of anisocoria.

In conclusion, pupillometry is a noninvasive, fast, and repeatable diagnostic method used in clinical ophthalmology, neurology, psychiatry, and research on autonomic system functioning. The clinical value of PLR assessment lies in its simplicity. PLR can now be measured using a mobile device, such as a smartphone equipped with an application, which has been shown to be non-inferior to the medical-grade pupillometer NPi-200. The broad accessibility of reliable pupillometry, especially in settings with limited access to advanced imaging requiring expensive diagnostic equipment, could significantly improve early diagnosis of ophthalmic conditions, such as glaucoma, and neurodegenerative diseases. This could even extend to primary care physicians equipped with such diagnostic tools.

## 5 Pupil size abnormalities

Under normal conditions, pupils are equally symmetrical. Any deviation is known as anisocoria (unequal pupil size). This condition can be caused by damage to iris autonomic innervation, both sympathetic (Horner’s syndrome) and parasympathetic (acute and chronic Adie’s syndrome), direct damage to the sphincter and dilator pupillae muscles (ischemia, angle closure glaucoma, iritis due to herpes zoster infection, trauma, and mechanical damage during anterior eye surgery), or pharmacological agent (anticholinergic and adrenergic mydriasis) influence.

To diagnose the muscles responsible for inefficient pupillary movements, inducing conditions that cause their activation is necessary. Damage to the sphincter pupillae intensifies anisocoria when pupils are exposed to light. If the abnormality involves the dilator pupillae, pupillary asymmetry will be more pronounced under low-light conditions.

Horner’s syndrome and physiological anisocoria should be differentiated in patients whose anisocoria intensifies under low-light conditions. In both cases, pupil asymmetry increases with light source weakening; however, the dynamics of this process are disturbed in the oculosympathetic defect.

### 5.1 Incidental anisocoria

Incidental anisocoria is a temporary (<12 h) pupillary asymmetry without accompanying neuro-ophthalmic symptoms. Patients may complain of blurred vision, problems with near visual acuity, or headaches. It is most common in adolescent and middle-aged women. Its causes are not fully understood; however, it is considered a symptom of vegetative system dysfunction (parasympathetic and sympathetic), often associated with migraine or epileptic seizures. Occasionally, the affected pupil may be elongated (tadpole pupil).

### 5.2 Physiological anisocoria

Approximately 20% of healthy individuals have a slight difference in pupil size of >0.4 mm, albeit no more than 1 mm. This is known as physiological anisocoria (simple anisocoria, mild anisocoria). It is more noticeable in dim lighting. In well-lit environments, about half of physiological anisocoria cases are not apparent.

Both pupils constrict normally when exposed to light. No delay is observed in pupil dilation when ambient light is turned off. Simple anisocoria may vary depending on the time of day and is observed in approximately one-fifth of the population. In some individuals, it usually occurs in the same eye. In others, it may be transient or occur alternately. Physiological anisocoria is unrelated to refractive error. These hypotheses assume transient asymmetric supranuclear inhibition of the Edinger–Westphal nuclei, resulting in an increased pupil size on the more inhibited side. They also explain why anisocoria decreases under light stimulation influence while looking at a near object, under anesthesia, during sleep, or under narcotic drug influence.

### 5.3 Horner’s syndrome

Horner’s syndrome develops slowly, mainly because of damage to the oculosympathetic nerve pathway (Castro et al., 2023). It is caused by denervation of the sympathetic nervous system, which may occur in the first-order (central), second-order (preganglionic), or third-order (postganglionic) sympathetic innervation neurons (Go et al., 2020). Damage can occur at three levels: (1) in the central part from the posterior hypothalamus through the brainstem to the spinal cord at the C8-Th2 level; (2) in the preganglionic part to the superior cervical ganglion; (3) in the ganglionic part from the superior cervical ganglion, through the cavernous sinus to the orbit (Bellégo et al., 2022; Hassold et al., 2022; Tang et al., 2022; Uludağ et al., 2017; Yoshida and Kawabata, 2018). Congenital or spontaneous changes of unknown origin may also cause this syndrome (Salman, 2024).

The clinical picture of Horner syndrome includes a triad of symptoms (ptosis, miosis, and enophthalmos), accompanied by other clinical changes, such as heterochromia, decreased sweating, and slow pupil dilation. Congenital or spontaneous changes of unknown origin may also cause this syndrome (Salman, 2024).

Ptosis is never severe because the elevating muscles of the upper eyelid are innervated by CN III. Symptom severity varies depending on whether the patient is tired or agitated. Ptosis usually increases in the evening and is less severe in the morning. Flaccidity of the upper eyelid skin can be difficult to diagnose in older adults. In such cases, the position of the lower eyelid, a smooth muscle tissue with sympathetic innervation, is of great importance. To assess their condition, patients are instructed to look at a light source held in their hands, which is moved upward. The procedure continues until the corneal limbus at the 6 o’clock position on the side affected by Horner’s syndrome barely touches the free edge of the lower eyelid. Subsequently, the other side of the eye is observed, and a fragment of the sclera between the free edge of the lower eyelid and the corneal limbus can be seen.

Miosis is excessive constriction of the pupil. Pupil dilation is distinctly slower when the light source is removed. Under normal conditions, both pupils dilate simultaneously. However, in myosis, the dilation reflex on the side affected by the pathology is delayed. Patients with this syndrome have weakened dilator pupillary muscles, resulting in much slower pupil dilation than normal. If the sympathetic defect is advanced, the affected pupil dilates only by the relaxation of the sphincter pupillae muscle. This process lasts longer than that achieved by activation of the dilator pupillae muscle. When the light source is turned off, anisocoria intensifies and becomes most severe in the 4th to 5th second. It begins to decrease after 10–20 s owing to continued sphincter pupillae relaxation through supranuclear Edinger–Westphal neuron inhibition.

Enophthalmos in Horner’s syndrome is subjective and results from a reduction in the elevation of the lower edges of the eyelids, which narrows the palpebral fissure.

Heterochromia refers to a difference in the coloration of the iris. At birth, the human iris is typically light blue or gray immediately after birth. The color usually changes to brown within the first year of life, depending on sympathetic innervation. This phenomenon is clearly visible in the eyes of patients with congenital Horner’s syndrome because the lack of sympathetic stimulation prevents the iris from darkening. The difference may be quite subtle and manifest itself only as a lighter shade of the iris in blue-eyed patients or very visible in patients with dark iris pigmentation in the healthy eye.

Other less common symptoms include conjunctival injection on the same side, reduced skin sweating, and reduced intraocular pressure on the same side. Intraocular hypotonia occurs when eye intraocular pressure on the side affected by Horner’s syndrome is at least 5 mmHg lower than that in the other eye.

Occasionally, lower eyelid elevation (inverted drooping) is observed. Other times, pain is observed on the side of the narrower pupil (mandibula, ear, and cheek) due to carotid artery dilation.

In the group of functional causes, no facial skin sweating disorders typical of this syndrome are observed because the damage to the pathway is located above the carotid artery branching. It usually affects men aged approximately 40 years. Headaches recur in waves, and Horner’s syndrome symptoms may persist. With cluster headaches, in addition to the previously mentioned typical symptoms, lacrimation, ocular congestion, and dryness of the nasal cavity mucosa occur on the affected side.

#### 5.3.1 Congenital and pediatric Horner’s syndrome

This condition originates from perinatal trauma or neuroblastoma. When a child has unilateral myosis and ptosis, the question arises whether it is definitively Horner’s syndrome (Silverman and Beres, 2022). In these cases, ptosis is moderately severe and never complete. Occasionally, inverse ptosis may be misleading. Children with congenital Horner’s syndrome may also exhibit weak, straight hair on the affected side if their hair is naturally curly, as hair follicle growth–like iris pigmentation–depends on adequate sympathetic innervation. Iris color is usually established between 9 and 12 months of age due to melanosome accumulation in melanocytes, which are innervated by sympathetic fibers. Therefore, if the pupil is poorly pigmented, heterochrony occurs, and oculosympathetic defects may develop early in life. However, it cannot be determined whether the origin is congenital or acquired. Diagnostic pharmacological tests with cocaine followed by phenylephrine should be performed to exclude pseudo-Horner’s syndrome (Martin et al., 2017). Additionally, asymmetrical facial flushing–redness on the healthy side and pallor on the affected side–may be observed when the child cries, although sweating asymmetry is often difficult to detect. Cycloplegia induced by atropinization may occasionally result in unilateral facial flushing, which can aid in diagnosis. Localization testing using hydroxyamphetamine is not recommended in infants due to the immaturity of the sympathetic nervous system. Given the potential association with neuroblastoma, Horner’s syndrome in children warrants further evaluation using imaging studies and urinary testing for tumor metabolites.

## 6 Pharmacological tests

### 6.1 4%–10% cocaine

The cocaine eye drop test facilitates Horner’s syndrome diagnosis with high precision (Li et al., 2022). The greater the anisocoria 50–60 min following administration, the higher the probability. If the difference in pupil size is at least 0.8 mm, the test is considered positive. Eye drops containing a 4% cocaine chloride solution (no more than two drops) are used for this purpose. This concentration is safe and does not cause adverse corneal reactions. The pH of the solution may cause substantial eye burning when applied to the eye surface. Cocaine blocks the reuptake of noradrenaline, which is normally released by the receptors. If noradrenaline is not released owing to sympathetic pathway disruption, cocaine does not produce an adrenergic effect. A pupil with Horne’s syndrome will dilate less under the influence of cocaine than a healthy pupil, regardless of the location of the defect. Furthermore, 45 min after cocaine drop administration to both eyes, anisocoria increases substantially, as healthy pupils dilate more than pupils with Horne’s syndrome. The effect of cocaine is based on the absence of pupilar dilatation on the affected pupil and the presence of dilatation on the healthy eye. Within 40–60 min after administration, anisocoria should be measured in a room with lighting. Slight eye dilatation with a suspected oculosympathetic defect and no pupil dilation even within 30 s in the dark before administration may lead to a false-positive result in the cocaine test. This situation can occur when myosis is caused by sphincter hyalinization or abnormal reinnervation of the sphincter pupillae, causing a pseudo-Horner’s syndrome (Brodsky, 2023). In such cases, administration of the directly acting sympathomimetic 2.5% phenylephrine at the end of a positive cocaine test will easily dilate the pupil suspected of a sympathetic defect, as cocaine-induced anisocoria should be eliminated. In some cases, phenylephrine causes even greater pupil dilation in patients with Horner’s syndrome because of hypersensitivity. Briefly, in pseudo-Horner’s syndrome, direct-acting sympathomimetic agents induce inadequate pupil dilation.

Currently, the cocaine test is rarely used and has largely lost its clinical significance, as it has been replaced by the apraclonidine test. However, it remains the recommended method in young children, since apraclonidine is contraindicated in this age group due to the risk of adverse effects such as bradycardia and central nervous system disturbances.

### 6.2 0.5% apraclonidine

**FIGURE 5:**
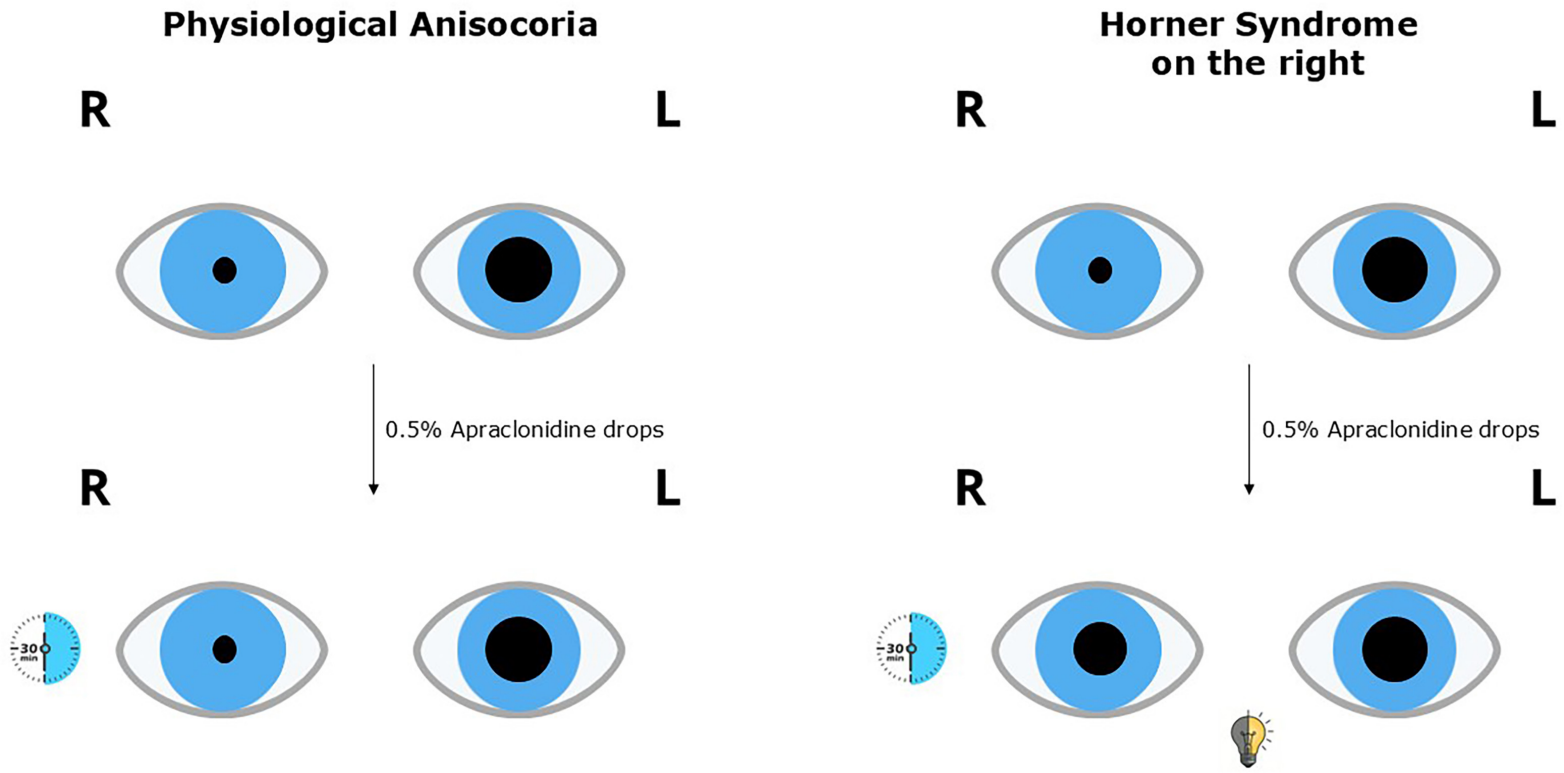
Apraclonidine test in physiological anisocoria and Horner syndrome.

Recently, the cocaine test was replaced with administration of 0.5% apraclonidine (Bremner, 2019). After apraclonidine administration to both eyes (30 min), the pupil with the oculosympathetic defect dilates, and the healthy pupil becomes slightly smaller in dim lighting, thereby eliminating or reducing anisocoria (Figure 5) (Jakobsen et al., 2023). In patients with anisocoria due to other causes, including physiologic anisocoria, pupil dilation is absent. Apraclonidine has an advantage over cocaine, as it actively dilates only the pupil with the defect. It also effectively penetrates the cornea more than phenylephrine, reaching high concentrations at iris receptors.

Determining the precise location of the sympathetic defects using pharmacological tests is also possible.

### 6.3 1% hydroxyamphetamine

Differentiating between Horner’s syndrome resulting from benign factors, such as vascular headaches, and more serious conditions, such as carotid artery dissection and preganglionic defects sometimes caused by neoplastic proliferation, is crucial (Clausen et al., 2023). Hydroxyamphetamine eye drops enable pathology localization in Horner’s syndrome, enabling precise targeting of radiological imaging (including lung apex and carotid artery). Occasionally, the changes associated with this syndrome are so characteristic that further determination of the damage is not necessary, such as, for instance, in patients with cluster headaches or after neurosurgical procedures that damage the oculosympathetic pathway in a given region.

Hydroxyamphetamine activates noradrenaline release from nerve terminals. When postganglionic damage occurs, most neurons die; therefore, noradrenaline is not stored at the nerve terminals. When the damage is severe, the pupil does not dilate in response to hydroxyamphetamine. This lack of dilation becomes evident at least 1 week after the injury, which corresponds to the time required for neuronal degeneration and depletion of stored norepinephrine. Consequently, performing this test before the lapse of that period may yield a false-positive result, suggesting a preganglionic location. Horner’s pupil, due to damage in a preganglionic or central location, will dilate to at least a normal size because noradrenaline is released from intact postganglionic storage sites despite the lack of synaptic connectivity. Moreover, in preganglionic neuron damage, the pupil on the affected side will dilate more than the healthy pupil due to “decentralization of hypersensitivity.” The principle of the test is as follows: pupil size is measured before and 40–60 min after hydroxyamphetamine drops administration into both conjunctival sacs. Changes in anisocoria are measured in a light room. If the pupil with Horner’s syndrome dilates less than the healthy pupil and the anisocoria increases by >0.5 mm compared with the state before administration, the lesion is postganglionic (along the carotid artery). If the smaller pupil dilates such that it becomes larger than the healthy pupil, the lesion is preganglionic, and the postganglionic neuron remains functional and releases noradrenaline. It is advisable to wait at least 2–3 days before performing a test using hydroxyamphetamine if performed after a test with cocaine, as this substance also blocks hydroxyamphetamine reuptake from postganglionic nerve endings, thereby blocking their action.

The hydroxyamphetamine test has lost its significance in differentiating between pre- and postganglionic Horner’s syndrome, as all patients with a confirmed diagnosis are now routinely referred for imaging of the head and neck. Moreover, in acute cases, waiting several days to perform this test is both impractical and not recommended.

### 6.4 Cholinergic hypersensitivity test with 0.1% pilocarpine

**FIGURE 6:**
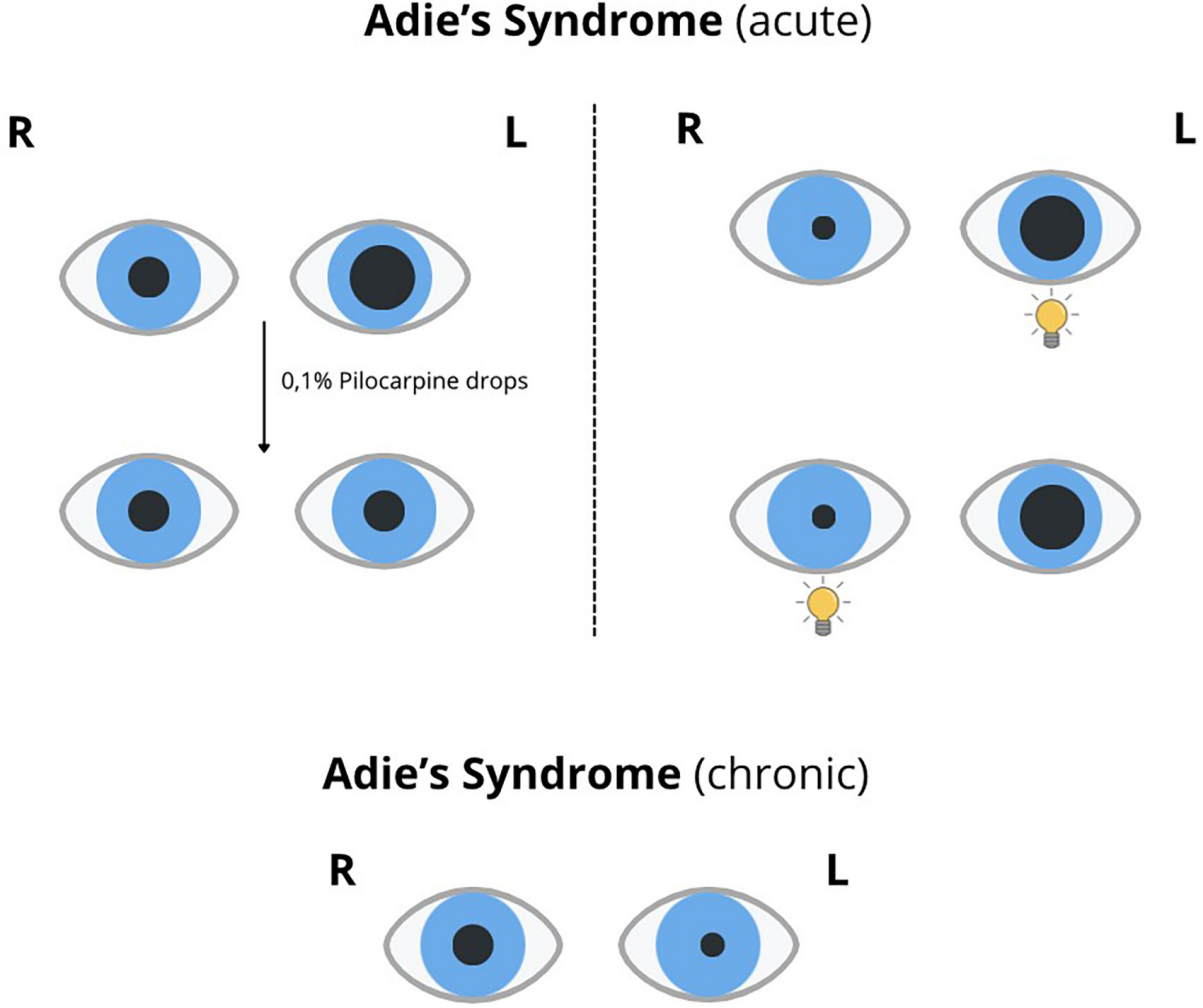
Adie’s syndrome: acute and chronic stages.

The cholinergic hypersensitivity test involves injecting a diluted 0.1% pilocarpine solution into both conjunctival sacs (Yoo et al., 2021). If the initially larger pupil constricts more than the normal-sized pupil following administration, this indicates sphincter pupillae hypersensitivity, suggesting partial loss of parasympathetic innervation. Such hypersensitivity reactions occur within 5–7 days of damage to the innervation. In postganglionic damage (either to the ciliary ganglion or to neurons distal to it), the sphincter pupillae exhibits more pronounced hypersensitivity compared to preganglionic lesions (e.g., CN III dysfunction). This reaction is characteristic of the tonically dilated pupil in Adie’s syndrome (Parajuli et al., 2022) (Figure 6). These reactions may be reversed depending on the phase of the syndrome. During the chronic period, when reinnervation occurs owing to increased accommodative cholinergic fibers, sphincter reinnervation may cause it to lose cholinergic hypersensitivity. Comparing the responses of both pupils is essential during testing, as there is great individual variability in cholinergic substance sensitivity.

## 7 CN III palsy

Pupil dilatation with impaired constriction occurs in response to light and near vision, accompanied by other ophthalmic symptoms such as eyelid drooping, impaired adduction, and downward or upward gaze. Occasionally, diplopia occurs. If the pupillary reflex to light is spared in the syndrome of damage to CN III, the damage is not caused by severe trauma or crushing but rather by a minor vascular disease, such as diabetes. Since the preganglionic parasympathetic nerves responsible for the light reflex are located in the middle section of the intracranial pathway of CN III and emerge from it in the midbrain, nerve compression in that location results in light reflex paralysis (Wilhelm, 2011). A common cause of that is an aneurysm (of the posterior communicating artery) or pituitary apoplexy (caused by sudden lateral expansion of a pituitary adenoma compressing the middle part of CN III). Pupil involvement is often incomplete; therefore, it is important to look for a mere weakening of its function due to anisocoria in bright light; only the sphincter segments may be paralyzed.

Occasionally, the third cranial nerve does not regenerate properly. It carries bundles that innervate various extraocular muscles (medial rectus, inferior rectus, inferior oblique, superior rectus, and levator palpebrae) and contains preganglionic parasympathetic nerves that go to the sphincter pupillae and ciliary body (Saju et al., 2023). Damage to CN III through the glial scaffold through which the individual nerve bundles pass stimulates nerve fiber regeneration, which can cause them to follow the wrong direction. For instance, when the patient looks downward, the eye may move abnormally, the pupil may constrict, or the palpebral fissure may widen due to atypical inward or outward turning of the eyeball. Abnormal regeneration can be primary or secondary. Secondary regeneration occurs approximately 8 weeks after sudden nerve damage. No sudden nerve damage is observed in primary regeneration. The nerve damage occurs slowly, through tumor growth or aneurysm enlargement, accompanied by abnormal regeneration with symptoms that gradually manifest.

## 8 Most common pupil shape abnormalities

Eyeball injuries often result in sphincter pupillae rupture or iris trabeculae damage, which are visible during retroillumination under a slit lamp.

A pinpoint pupil is observed because of severe eye injury, where a fragment of the iris protrudes through a wound that penetrates the ocular coat. If a blunt injury causes detachment of a piece of the iris from its base (iridodialysis), the pupil may become displaced and distorted. Such pupils also do not react normally to light (Kurimoto et al., 2011). The remaining reaction is the result of the proper functioning of undamaged iris segments. Mydriasis occurs due to siderosis or chalcosis in the presence of an intraocular metallic foreign body.

Post-traumatic iris paralysis may lead to pupil dilation (Wilhelm, 2011); the pupil appears dilated, usually oval or notched, due to damage to the stroma or iris sphincter muscle. The affected pupil exhibits impaired constriction in response to light and near vision. Patients typically report a history of trauma, intraocular surgery, or anterior segmental inflammation. Occasionally, rupture of the iris sphincter muscle, stromal atrophy, iris transparency changes, or adhesions can be observed. In some cases, angle recession signs are also visible (Legocki et al., 2023).

A widely dilated pupil, usually unilateral, with a sluggish reaction to light, may also occur because of head trauma, sometimes even minorly. Such cases are consequences of brain edema or intracranial hemorrhage of varying severity. This is particularly characteristic of severe head injuries, which are often accompanied by symptoms of concussion or loss of consciousness.

An irregular pupil (oval, clover-shaped, and abnormally sized–either constricted or dilated) may result from anterior segment inflammation, leading to the formation of anterior synechiae with the cornea or posterior synechiae with the lens surface (located just behind the iris). In the acute phase, this condition is accompanied by other signs of inflammation such as pain, eye redness, and decreased visual acuity. After herpes zoster infection, the sphincter muscle is often atrophic, with large geographic defects visible under retroillumination (Hanada et al., 2023). A pupil dilated into a specific vertical oval shape is characteristic of an acute angle-closure glaucoma attack, a particular form of this disease.

### 8.1 Holmes-Adie’s pupil (tonic pupil)

Tonic pupil most commonly occurs in young adults (more frequently in women aged 20–40 years) (Xu et al., 2022). The pupil is dilated and regular, with a very slow or absent reaction to light, especially in the presence of “vermiform movements” resulting from segmental sphincter pupillae deviation, with some segments still responding normally to light (Nene et al., 2021) (Figure 6). Slit lamp examination reveals segmental motility disturbances of the sphincter pupillae muscle and increased iris translucency (iris atrophy). Corneal sensitivity may also be reduced in the affected eye; however, no other abnormalities are found on ophthalmological examination. During the first week, cholinergic hypersensitivity is present. After 2 months, the condition progresses to a chronic stage. Nerve fibers begin to proliferate, and neurons initially connected to the ciliary muscle begin extending into the sphincter pupillae (Xu et al., 2022). The light response of the denervated segments does not return to normal; however, these segments begin to properly contract when focusing on nearby objects, leading to a dissociation between light and near responses, along with partial accommodative ability restoration. Pupil constriction in response to near vision is tonic, whereas dilation upon shifting the gaze to a distant object occurs very slowly. Although some accommodative functions are restored, the responses remain weak and slow. Patients often report difficulty in maintaining visual clarity when shifting focus between near and far objects. Eventually, the affected pupil becomes smaller, especially under dim lighting, due to the overgrowth of accommodative cholinergic fibers, which keep the sphincter in continuous contraction.

Some patients also lack normal reflex responses to sudden stimuli and have reduced vibration sensations, suggesting similar pathological processes in the medulla oblongata. This is due to damage to the ganglia of the dorsal root, leading to abnormal deep tendon reflexes. Coexisting tonic pupil and weakening of deep muscles reflexes is called Adie’s syndrome. However, Adie’s syndrome is not associated with severe neurological disorders or significant dysfunctions. The exact cause remains unknown; however, some hypotheses suggest an autoimmune defect (Yin et al., 2023). It can occur in children following mumps infection. After 10 years, 50% of patients develop similar symptoms in the second eye.

Holmes-Adie’s pupil is usually caused by damage to the ganglionic nerve fibers (ciliary ganglion or ciliary nerves) that innervate the sphincter pupillae, most often due to a viral infection. Other rare causes of the tonic pupil include retinal laser photocoagulation, tumors, trauma, orbital surgery, and diffuse neuropathies, such as the Guillain–Barré syndrome, Riley–Day syndrome, congenital neuropathies, amyloidosis, syphilis, and generalized autonomic dysfunction.

In the chronic phase, it can be challenging to differentiate between the dissociation of the reaction to light and near vision present in tonic pupils and the symptoms present in Argyll Robertson pupils. In Holmes-Adie’s pupil, the affected pupil shows minimal constriction in response to light but improves with accommodation (near response). In contrast, in Argyll Robertson pupils, reaction to light is absent, while the near-vision reaction is preserved.

The pharmacological Holmes-Adie’s pupil recognition test involves administering a weakly concentrated solution of pilocarpine (0.125%) or a 2.5% solution of methacholine (ClinicalGate, 2015). After administration of the drug, the tonic pupil constricts, while there is no reaction from the normal pupil. This is explained by denervation hypersensitivity.

## 9 Effect of medication on pupil size

Pupil size is considerably influenced by medication, whether applied topically to the conjunctival sac or administered systemically via tablets or injections (Halámková et al., 2021; Ruiz-Barrio et al., 2022). The following are pupil-dilating agents, regardless of the form of administration: atropine, cyclopentolate (tropicamide), homatropine, and epinephrine. Conversely, pilocarpine, carbachol, echothiophate, demecarium, physostigmine, neostigmine, fluostigmine, aceclidine, acetylcholine, and paraoxon lead to pupil constriction. Systemic drugs that induce pupil dilation include cocaine and marijuana, whereas morphine causes constriction (Soulis et al., 2022; Zhang et al., 2022).

## 10 Collaboration with other specialists

Although such collaboration is not always fully implemented, it is indispensable for diagnostically difficult cases. Most general examinations, especially brain imaging, are recommended by consultants from other specialties, such as neurologists, neurosurgeons, ENT specialists, and maxillofacial surgeons.

### 10.1 Discussion

This document is a comprehensive review article on pupillary disorders, their diagnosis, and implications in various medical fields. The key significance and implications presented in the document are:

### 10.2 Diagnostic value in neurology and ophthalmology

Pupillary disorders play a crucial role in diagnosing and monitoring various neurological and ocular conditions. The pupil’s response to light and near objects, as well as its size and symmetry, can provide valuable information about the functioning of the nervous system and the presence of certain diseases.

### 10.3 Interdisciplinary relevance

The study of pupillary disorders has implications beyond ophthalmology, extending to neurology, psychiatry, and other medical specialties. Pupillary abnormalities can be indicators of systemic diseases, autonomic nervous system dysfunction, and even early signs of neurodegenerative disorders.

### 10.4 Advancements in diagnostic techniques

This review highlights the importance of modern diagnostic methods like pupillometry, which allows for more precise and objective assessment of pupillary function. These advanced techniques enable the detection of subclinical changes and early symptoms of various diseases, potentially leading to earlier interventions and better patient outcomes.

### 10.5 Pharmacological testing

The article discusses various pharmacological tests used in diagnosing specific pupillary disorders, such as Horner’s syndrome. These tests help in localizing the site of damage in the sympathetic pathway, which is crucial for appropriate treatment planning.

### 10.6 Implications for patient care

Understanding pupillary disorders and their underlying mechanisms can lead to improved patient care across multiple medical specialties. This knowledge aids in more accurate diagnoses, better monitoring of disease progression, and potentially more targeted treatments for conditions affecting the autonomic nervous system and visual pathways.

In conclusion, this review underscores the importance of thorough pupillary examination and the potential of advanced pupillary assessment techniques in enhancing diagnostic accuracy and patient care across various medical fields.

### 10.7 Limitations

The existing literature on pupillary disorders is extensive in terms of anatomical and physiological descriptions, yet it remains fragmented across subspecialties. Foundational texts and classical studies provide a robust framework for understanding basic pupillary reflex pathways, but they often lack integration with modern diagnostic techniques such as dynamic and chromatic pupillometry. Moreover, while many case reports and clinical observations describe pupillary abnormalities in isolation, few studies offer a systematic or interdisciplinary perspective that connects ocular findings with neurological and systemic pathologies.

Recent publications have advanced the field by highlighting the diagnostic value of pupillometry in conditions like Parkinson’s disease, Alzheimer’s disease, glaucoma, and diabetic autonomic neuropathy. However, many of these studies are small-scale, observational, or lack standardization in methodology. The absence of consensus protocols for pupillometric parameters (e.g., light intensity, wavelength, adaptation time) limits cross-study comparability and reduces the generalizability of findings. Additionally, while ipRGC-mediated responses and PIPR have been proposed as promising biomarkers, large-scale validation studies are still lacking.

The pharmacological testing literature is well-established but remains underutilized in routine clinical settings. Emerging comparisons between agents such as cocaine, apraclonidine, hydroxyamphetamine, and pilocarpine provide valuable diagnostic insights, particularly in Horner’s syndrome and Adie’s tonic pupil. Nevertheless, inconsistent availability of certain agents and variation in interpretation criteria limit their practical application.

Furthermore, while there is growing interest in the application of pupillometry in psychiatry and critical care, the supporting evidence remains preliminary. In these domains, pupil dynamics are increasingly being explored as proxies for autonomic tone, emotional reactivity, or intracranial pressure, but such associations need rigorous longitudinal validation.

## 11 Conclusion

Pupil examinations are crucial in the diagnosis and monitoring of many diseases, and technological advancements in the field of pupillometry allow for more accurate pupil function analysis. Modern diagnostic methods, such as pupillometry and dynamic pupillometry, allow for a precise assessment of not only pupil function but also the structures of the central nervous system, while pharmacological diagnostic tests, such as with cocaine or apraclonidine, enable precise determination of the damage location to the sympathetic system. Future research focusing on the mechanisms of pupil regulation may contribute to a better understanding of ophthalmological pathologies, as well as neurological and systemic diseases. In conclusion, while the literature provides a strong foundational understanding and promising clinical applications of pupillary assessment, it suffers from methodological heterogeneity and insufficient interdisciplinary integration. There is a need for larger, standardized, and prospective studies to validate pupillary metrics as reliable biomarkers and to formalize their use across diverse clinical contexts. This review contributes by consolidating dispersed knowledge and emphasizing the need for a unified, clinically applicable framework for pupillary diagnostics.
